# When the Evidence Points to the Non-Invasive Nature of an Allegedly Invasive Alien Species: The Case of the Aoudad in Mainland Spain

**DOI:** 10.3390/ani15182683

**Published:** 2025-09-13

**Authors:** Jorge Cassinello, Elena Albanell, Sergio Eguía, Andrea Roverso, Alfonso San Miguel, Jordi Bartolomé

**Affiliations:** 1 Experimental Station of Arid Zones, Spanish National Research Council (EEZA, CSIC), Carretera de Sacramento s/n, La Cañada de San Urbano, 04120 Almeria, Spain; 2 Department of Animal and Food Sciences, Facultat de Veterinària, Universitat Autònoma de Barcelona, Travessera dels Turons s/n, Bellaterra, 08193 Barcelona, Spain; elena.albanell@uab.cat (E.A.); andrea.rov1996@gmail.com (A.R.); jordi.bartolome@uab.cat (J.B.); 3 Mendijob S.L., C/Rambla 22, El Palmar, 30120 Murcia, Spain; sergio.eguia.martinez@gmail.com; 4 Departamento de Sistemas y Recursos Naturales, E.T.S. Ingeniería de Montes, Forestal y del Medio Natural, Universidad Politécnica de Madrid, C/ José Antonio Novais 10, Moncloa-Aravaca, 28040 Madrid, Spain; alfonso.sanmiguel@upm.es

**Keywords:** alien species, *Ammotragus*, biodiversity conservation, biological invasions, ecological knowledge, invasive species, restoration ecology, trophic ecology, vacant niches, wildlife

## Abstract

Here we reveal a first comparative analysis of the use of food resources of wild populations of the aoudad *Ammotragus lervia* and the Iberian ibex *Capra pyrenaica*, both in sympatric and allopatric conditions. The study was carried out in the southeastern mountains of the Iberian Peninsula, in the Murcia region. Our results show that the aoudad preferentially behaves as a grazer herbivore, the herbaceous component being present in its diet by 57%. The ibex, on the contrary, shows a 63% preference for shrubs. When in sympatry, the ibex shows a comparatively higher incidence on the herbaceous stratum. In summer, the aoudad’s consumption of woody species increases. The differences observed in the feeding behavior of ibexes and aoudads most probably make their joint presence in the study mountainous ecosystems compatible, provided sufficient resources are available. Ecological implications and management proposals are discussed in the context of alien species management.

## Simple Summary

This study compares the feeding habits of two wild mountain-dwelling herbivores: the aoudad (*Ammotragus lervia*) and the Iberian ibex (*Capra pyrenaica*), in the mountains of southeastern Spain. In some mountain ranges within the study area, populations of both species coexist, while in others, only one of the two is present. This has allowed us to conduct a comparative study depending on whether they share the same space or not. Understanding how they share food resources is important for managing natural areas, especially when one of the species, the aoudad, is not native to the region. The results showed that aoudads mainly eat grasses and other herbaceous plants, while Iberian ibexes prefer shrubs. However, when both species live together, ibexes eat more herbaceous plants than usual, and in summer, aoudads increase their intake of woody plants. These differences in diet suggest that both species can coexist in the same environment if there is enough food available. These findings offer valuable information to understand aoudad feeding habits and their potential competition for resources with the native Iberian ibex, and thus empirically clarify whether their ecological role can certainly be considered invasive, an essential issue for proper management of their populations.

## 1. Introduction

The study of invasive alien species has become increasingly important in recent years, being currently considered as one of the major threats to native species and biodiversity conservation [1,2]. Invasive alien species are defined as species that have been introduced to a new environment and have established self-sustaining populations, where they can cause harm to the environment, economy, or human health. They can outcompete native species for resources such as food, water, and shelter, and often have no natural predators in their new environment. This can lead to a decline in native species populations and even extinctions.

The case of the aoudad (*Ammotragus lervia*) free-ranging populations present in mainland Spain is particularly controversial, see [3]. Contrary to the European mouflon *Ovis aries musimon*, the other alien ungulate introduced in the wild in Spain [4], the aoudad was included in the Spanish List of Invasive Alien Species by the Real Decreto 630/2013, as it has been considered harmful to the environment.

The aoudad is a North African caprid that originally inhabited most of the arid and semiarid lands of the Sahara and Sahel regions [5]. It is currently under a Vulnerable status in its native lands, according to the IUCN Red List [6], with a distribution characterized by isolated and scattered populations. Their populations have been drastically reduced due to uncontrolled hunting, changes in their habitats, and competition with domestic cattle [6]. Outside its native range, it has been introduced in different countries as a game species in fenced hunting estates, but on several occasions accidental escapes have given rise to free-ranging stable populations [7,8].

In Spain, the species was introduced in 1970 in the Sierra Espuña (Murcia), as a new game species and where no other wild ungulate was present at that time, see [9]. Two years later, it was also introduced in La Caldera de Taburiente National Park (La Palma Island, Canary Islands). Also, at the beginning of the 90s, there were escapes from two hunting estates in Alicante province, which gave rise to another stable population [10]. The population introduced in Murcia is a particularly relevant example, due to its success, its rapid growth, and the expansion developed over an area of more than 4400 km^2^.

The colonizing capacity of the aoudad in southeastern Spain and its potential threat to native species have been called to attention [11]. One of the greatest concerns referred to the potential damage the aoudad could cause to the populations of the Iberian ibex, *Capra pyrenaica*, the Iberian native ungulate closest to the aoudad phylogenetically, and therefore, at a behavioral and ecological level. A comparative study of ecological niche preferences for both ungulates showed strong similarities, although detailed analyses revealed a greater preference of the aoudad for more arid lands, with less tree cover, higher summer temperatures, and lower winter precipitation rates compared to Iberian ibex preferences [12]. Subsequently, a comparative study of diet selection by several ungulate species in sympatry indicated that the aoudad preferentially selected herbaceous species, showing a marked grazing tendency [13]. In comparison, the Iberian ibex preferentially shows a browsing behavior [14]. The same feeding pattern has been observed in native aoudad populations from Tunisia [15], Algeria [16], and Morocco [17], where there appears to be a prevalence of grasses and forbs in their diet.

Regular monitoring of the presence of aoudads and Iberian ibexes in the province of Murcia has allowed for the recording of a gradual colonization process of mountainous areas also by the ibex, which on occasion seems to displace aoudads from some mountains [18]. Before the arrival of these wild caprids, the study area had an intense livestock activity, with up to 10,000 goats and sheep counted in the Sierra Espuña alone [19]. Finally, recent studies on woody plant preferences by both herbivores in southeastern Spain showed no differences in their incidence on threatened plants, which was herbivore density dependent, regardless of their origin [20,21].

Although to date there is not a single evidence that shows negative effects of the aoudad on the host ecosystem in its distribution in Iberian areas, associated with its exotic origin, the species is included in the Spanish List of Invasive Alien Species, largely due to a misinterpretation of the invader concept, see [3].

This study provides the first empirical information, scientifically supported, that allows us to determine the comparative incidence of the aoudad and the Iberian ibex, under allopatric and sympatric conditions, on the herbaceous and woody strata in the southeastern Iberian Peninsula. This comparative incidence will allow us to examine scenarios of competition for resources between both species, as well as the level of impact that the alien species has on this Mediterranean ecosystem.

## 2. Materials and Methods

### 2.1. Study Sites

**Figure 1:**
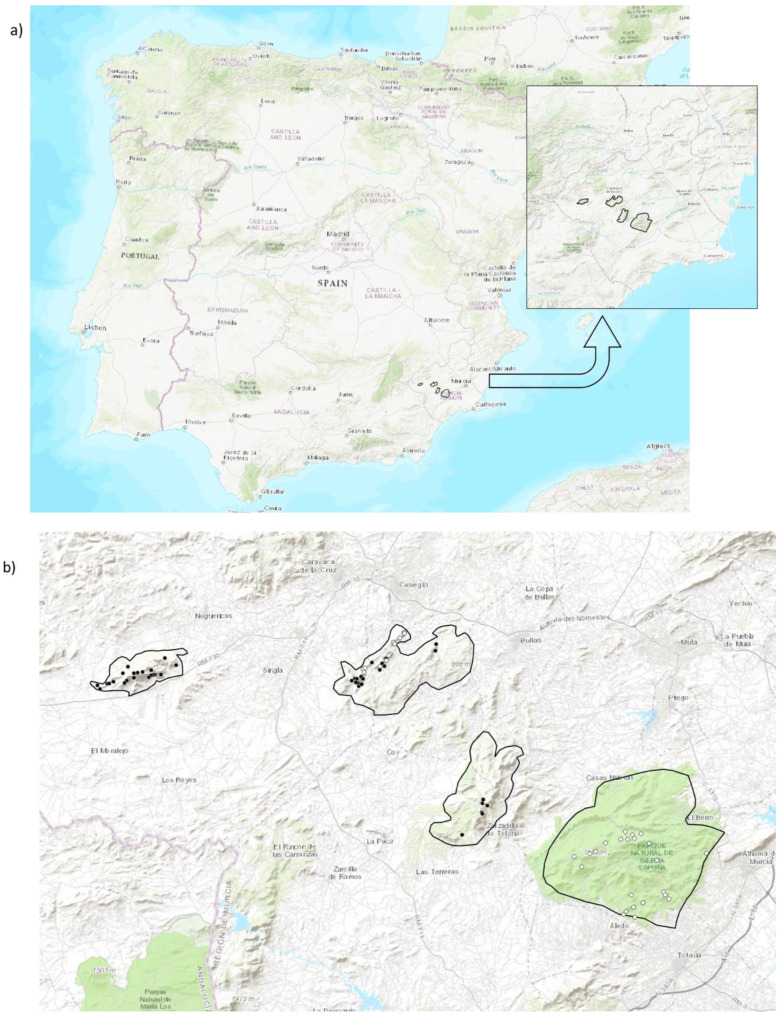
Delimitation of the study area. (**a**) Localization in Murcia province (inset image), southeastern Iberian Peninsula. (**b**) The four study areas and sampling points were ibex (black dots) and aoudad (white dots) feces were collected. Mountains from left to right: Mojantes/Caravaca (only ibexes), Burete/Quipar/Cabras and Cambrón (ibexes and aoudads), and Espuña (only aoudads). Basemap sources: © OpenStreetMap contributors (ODbL) and © Esri, accessed via QGIS 3.12.1.

A previous survey of the mountain ranges where aoudads and Iberian ibexes are present in the Region of Murcia allowed us to establish the optimal areas to carry out a comparative study of their trophic behavior. The mountains chosen for this study have similar habitats and plant communities, being representative of the Mediterranean ecosystem of southeastern Spain. In addition, we selected mountains that, at the time this study was undertaken, were either inhabited solely by stable ibex or aoudad populations or by populations of both species in sympatry (Table 1). No other wild ungulate was present in the study sites, apart from wild boar *Sus scrofa*, and only occasionally, scattered and small herds of domestic livestock were observed, but in very localized and known localities, so that their incidence on the herbivore community could be considered residual and negligible with regard to our analyses. All the mountain ranges chosen are located to the west and northwest of the Sierra Espuña mountains (see Figure 1), also included in this study, and where the original introduction of the aoudad took place in 1970 [22].

### 2.2. Sample Collection

Seasonal fecal samples were collected from the two herbivore species under study, both under sympatric and allopatric conditions. Hence, from November 2020 until August 2021, four field campaigns were carried out, one per season. In each campaign, all the study sites were visited on foot early in the day, in search of groups of aoudads and/or ibexes. Once a group was located and registered, we approached where they were observed in search of fresh feces, which were later collected and stored in properly labeled zip bags. Each sample consisted of 10 pellets. Twenty samples were collected from each animal species in sympatry and allopatry during each season, resulting in a total of 320 samples. The samples were preserved frozen until they were taken to the laboratory for further analyses.

### 2.3. Laboratory Analyses

To carry out the analysis of the trophic behavior of the study species, we estimated the plant species consumed through two laboratory techniques: the microhistological analysis of fecal remains through the identification of plant cuticles at the microscope following the protocol described in [23] and adapted according to [24], and the subsequent calibration of this procedure to apply the near-infrared spectroscopy (NIRS) methodology [25].

Microhistological analysis has been carried out on 64 fecal samples, distributed in 39 of aoudad and 25 of ibex, collected in the four seasons of the year and in different mountains of Murcia, depending on whether the species are in sympatry or allopatry. They have counted 100 identifiable plant fragments in each sample. The different plant fragments were grouped into two categories: herbaceous (forbs and grasses) and woody species.

The identification of epidermal fragments was based on the shape of epidermal cells, trichomes, and stomata surrounding cells, and a photographic repository of plant epidermis was used (available on the website: https://ddd.uab.cat/collection/atlepi?ln=es accessed on 10 June 2025). Epidermal fragments of floral parts, stems, petioles, and midribs were not registered due to difficulties in species identification.

The identification of plant fragments, without reaching the genus or species level, was based on epidermal features that allow differentiation between herbaceous and woody Mediterranean species:

(a) Graminoid herbaceous plants. Very long, rectangular epidermal cells, often interspersed with short, silicosuberous cells. Stomata are of the graminoid type, with bone-like guard cells. Trichomes are spine-like, resembling rose thorns.
(b) Other herbaceous plants. Rounded or lobed epidermal cells with very thin walls, typical of annual species with a thin cuticle. Stomata possess kidney-shaped guard cells. Trichomes may be long and slender, needle-shaped, either unicellular or segmented conical (always unbranched), or unicellular and star-shaped.
(c) Woody plants. Polygonal or rounded cells, rarely lobed, with very thick walls, are typical of evergreen species with a thick cuticle. Stomata also have kidney-shaped guard cells. Trichomes may be short and thick, needle-shaped, multicellular and star-shaped, or segmented and branched.

The microhistological analysis was performed at the Animal Production Laboratory of the Veterinary Faculty of the Universitat Autònoma de Barcelona (UAB). This laboratory participates in the ASFAC-LAB quality program, which involves an interlaboratory comparison exercise for the analysis of feed and raw materials used in animal nutrition (https://www.asfac-lab.com/en/ accessed on 10 June 2025).

The potential of fecal NIRS for estimating the composition of major plant groups has already been demonstrated using fecal cuticle microhistological analysis as the reference method [25]. In this study, fecal samples for NIRS analysis were ground to 1 mm and scanned with a NIRSystems 5000 scanning monochromator (FOSS, Hilleröd, Denmark). Reflectance was recorded in 2 nm steps, from 1100 to 2500 nm, which gave 692 data points for each sample, as log (1/R), where R represents reflected energy. The samples were scanned in duplicate using closed-ring cup cells, and the mean spectrum was calculated for each sample. The calibration process was performed according to the procedure described in [25].

### 2.4. Statistical Analyses

A parametric ANOVA test was carried out through a standard least squares fit model. The response variable used was the proportion of woody plants present in the diet, whereas the explanatory variables were Species (aoudad, ibex), Distribution (sympatric, allopatric), Season of the year, and their crosses (Species × Distribution, Species × Season, Distribution × Season, Species × Distribution × Season). The arcsine square root transformation was made to accomplish normality, and the significance level was established at 0.5. Thanks to the potential of NIRS to predict the diet composition, we increased the sample size fivefold (from 64 to 320 samples), which increased the statistical power of the test applied. The statistical program used was JMP^®^ 8.0.2. for Apple MacOS.

## 3. Results

Table 2 and Table 3 show the botanical composition of ibex and aoudad diets obtained, respectively, from the microhistological analysis of fecal content and the fecal NIRS predictions. The resulting variance model performed was highly significant: F(15,318) = 14.8742, *p* < 0.0001, both for the individual explanatory variables and their respective crosses.

Table 4 summarizes the ANOVA Effects Test for the proportion of woody plants. The factor Species had the strongest effect (F = 109.99, *p* < 0.0001), indicating clear trophic differences between the alien aoudad and the native Iberian ibex. Distribution (allopatry vs. sympatry) also had a significant but weaker effect (F = 4.41, *p* = 0.0366), while Season showed a marked influence (F = 15.64, *p* < 0.0001). Several interactions were significant as well, particularly Species × Distribution (F = 11.69, *p* = 0.0007), Species × Season (F = 3.56, *p* = 0.0147), Distribution × Season (F = 4.96, *p* = 0.0022), and the three-way interaction Species × Distribution × Season (F = 8.11, *p* < 0.0001).

**Figure 2:**
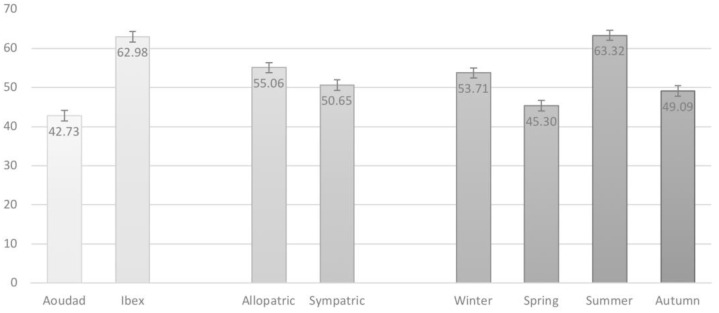
Average of the proportion of woody plants (± SE) present in the diet of the study herbivore species, according to three explanatory variables (species, distribution, and season of the year).

Figure 2 illustrates the main effects. The ibex consistently showed a higher proportion of woody plants in its diet (62.98%) compared with the aoudad (42.73%), supporting the strong species effect detected in Table 4. In terms of distribution, individuals in allopatry consumed more woody species (55.06%) than those in sympatry (50.65%). Seasonal differences were also evident: woody consumption peaked in summer (63.32%) and winter (53.71%), while spring (45.30%) and autumn (49.08%) showed lower values.

**Figure 3:**
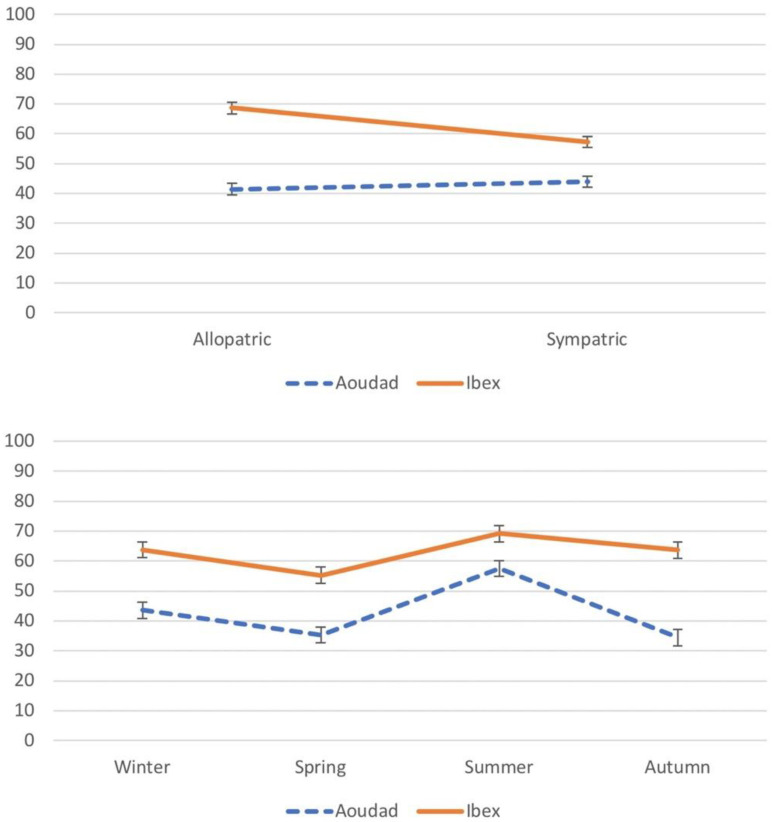

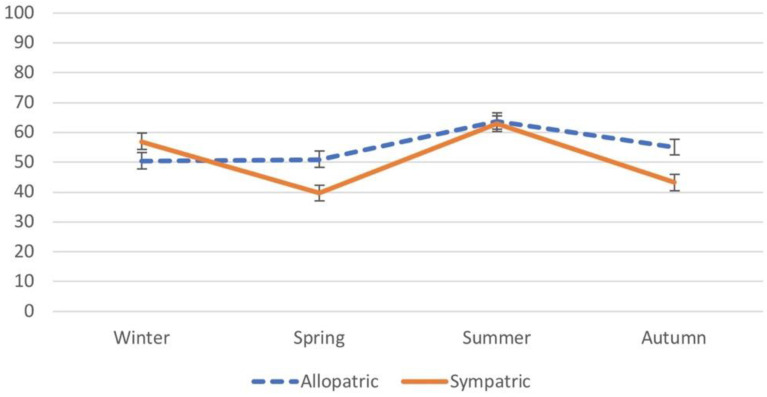
Average of the proportion of woody plants (±SE) present in the diet of the study herbivore species, according to the pair crosses of the three explanatory variables, species x distribution (upper), species × season (middle), and distribution × season (lower).

Figure 3 further details the significant interactions. The Species × Distribution plot (upper panel) shows that ibex reduced woody consumption in sympatry (from 68% to 58%), whereas aoudad maintained similar values across distributions (~43%). The Species × Season interaction (middle panel) reveals that both species increased woody intake in summer, but the ibex maintained consistently higher values than the aoudad across all seasons. Finally, the Distribution × Season interaction (lower panel) highlights that differences between allopatric and sympatric populations were especially marked in summer and autumn.

Overall, these results confirm significant trophic segregation between species: the aoudad behaves predominantly as a grazer, with ~57% herbaceous plants in its diet, while the ibex shows a clear preference for woody plants (~63%). Distributional context and seasonality modulate this pattern, with the ibex slightly increasing herbaceous intake in sympatry and both species raising woody consumption in summer.

## 4. Discussion

A comparative diet analysis of two herbivore species of different origins, but inhabiting the same area, is shown here. This study is particularly relevant due to the fact that it is dealing with a native species, the Iberian ibex, and an alien species, the aoudad, which is considered invasive by the authorities. It is evidenced that 57% of aoudad’s diet comprises grasses and forbs, whereas the ibex, on the contrary, shows 63% of woody components. When in sympatry, the ibex shows a comparatively higher incidence on the herbaceous stratum. In summer, the aoudad’s consumption of woody species increases.

### 4.1. Aoudad Invasiveness in Question

According to the International Union for Nature Conservation (IUCN) and the Convention on Biological Diversity (CBD), alien invasive species are those that threaten the biodiversity of the host ecosystem and/or cause serious economic damage [26,27]. The aoudad, a caprid originally from North Africa, has been widely introduced elsewhere [7,8], including Spain, where it is considered invasive by the Spanish authorities (Real Decreto 630/2013).

A recent paper [3] pointed out that empirical evidence does not show so far any deleterious effects of the aoudad on the host Iberian ecosystems where it was introduced since 1970. However, its colonizing character and relatively high reproductive rate raised deep concern about its potential as a serious competitor for resources with native ungulate species, along with its impact on native vegetation [11,12]. Nevertheless, even though the hypothesis on the aoudad having negative impacts on the host ecosystem was quite plausible, field studies were needed to corroborate this issue, see [3].

As research on the ecology of the aoudad in Iberian lands was carried out, a series of a priori unexpected facts emerged:

- It is a species with a marked grazing behavior, even higher than that of a wild sheep such as the European mouflon [13].
- It has an incidence on protected woody plant species similar to that of native species such as red deer *Cervus elaphus* and the Iberian ibex [21]
- Contrary to previous alarming expectations [11], we appreciate that its expansion potential has not been reached after more than half a century since its introduction. The reasons for that may lie in constraints to aoudad population dispersal associated with the intensity of human disturbance and land use [11]. Also, recent eradication attempts undertaken by the Murcia regional authorities resulted in an important decrease in their population size [3].
- In the present work, substantial and significant differences between aoudad and Iberian ibex trophic behavior are shown, suggesting a new more plausible hypothesis on a viable coexistence between both herbivore species [28], and furthermore, a positive ecosystemic role played by the aoudad, by markedly influencing the herbaceous layer and therefore facilitating the existence of clearings in forest lands and greater biodiversity in natural pastures [29,30]. In contrast, comparative feeding studies between sympatric aoudad and wild sheep species (i.e., the European mouflon [13] and desert bighorn sheep [31]) have reported greater diet overlap, which may indicate a greater potential for trophic competition between these species.

### 4.2. Comparative Trophic Incidence of Aoudads and Ibexes

One of the peculiarities of this study is that the trophic behavior of the aoudad has been compared with that of the native Iberian ibex both under conditions of coexistence (sympatry) and in the absence of coexistence (allopatry). This has allowed for the first time to empirically verify whether resource partitioning or competition is expected between these two ungulate species.

The results obtained confirm previous studies on the food preferences of the aoudad, both in its introduced populations [13] and in those present in its native distribution areas [15,16,17], highlighting a grazing character, that is, preferentially selecting herbaceous plants (grasses and forbs). In contrast, the ibex generally shows a marked preference for woody species in its diet, as has been observed in other Iberian populations (see a review in [32]).

However, we have shown a significant change in the feeding habits of the ibex when it is in sympatry with the aoudad (Figure 3, upper panel), choosing in this case a higher percentage of herbaceous plants. This unexpected result, in which the native herbivore changes its feeding habits compared to those chosen under allopatric conditions, opens up a new scenario of interaction between species that is worth commenting on, and for which we have no more than new hypotheses that would be worth testing in future studies.

1. A first hypothesis would suggest the formation of mixed groups of both species as an adaptive defense strategy against predators [33], where the smaller species follows the path and thus the feeding sites selected by the larger and stronger species. Regarding the ungulates studied, the average body weight of adult aoudad males may be more than 50% greater than that of Iberian ibex males, 82 kg vs. 50 kg, according to [34,35], respectively.
2. Another hypothesis would be related to the selection of highly nutritious food [36]. In sympatry, populations of both species may coincide in time and space, and as the aoudad tends to preferentially graze on good pastures, a hypothetical explanation of the higher selection of herbs by the Iberian ibex, a more opportunistic herbivore, when in sympatry, may lie on the fact that aoudads are able to nutritionally exploit grasses and forbs as they move through their grazing paths, and this, in turn, provokes a compensatory regrowth that may be exploited by the ibex. We do not consider that this greater dietary overlap under sympatric conditions supports an increase in resource competition. On the contrary, what we observe is a trophic shift in the diet of the Iberian ibex as a consequence of the presence of the aoudad—a selective change that is not related to differences in pasture availability in those areas, since all study sites share a very similar vegetation mosaic structure.

As evidence of this trophic interaction, it should be noted that during the field sampling of this project, in the areas of sympatry, individuals of both species were observed on numerous occasions feeding nearby (S. Eguía, pers. obs.).

Diet seasonal differences have also been noticed. Thus, the percentage of woody plants in the diet of both species is higher during the summer season, when the availability of herbaceous plants diminishes significantly due to the Mediterranean summer drought. It is worth mentioning here that shoots and leaves of woody plants can be—and in the Mediterranean often are—of higher quality than graminoids, especially during summer [37].

Furthermore, as these summer draughts may become more intense under the current global warming scenario [38], the species more adapted to semiarid conditions will be in a more advantageous position, particularly non-native ones [39]. Indeed, the aoudad is not only adapted to intense drought periods and high average temperatures in its native North African lands, but habitat suitability models have already shown its preference for these climatic features in southeastern Spain [11,12]. This suggests that the exotic aoudad might be better adapted than most native ungulates to the predicted climate change scenario [31,40].

### 4.3. Ecological Implications and Management Proposals

The marked grazing character of the aoudad, along with an evidenced incidence on the shrub layer similar to that observed in other native Iberian ungulates [21] present it as a potentially key actor to control and restrict current processes of shrub encroachment, maintaining natural grasslands, particularly in arid zones, many of them included in the Natura 2000 Network, see [40]. Also, the presence of a wild grazer such as the aoudad can compensate for the lack of grazing herbivores in the study area, see [19].

In any case, and as it is also established for native ungulate species, a sustainable presence of the aoudad in southeastern mainland Spain must necessarily imply the control and monitoring of their populations, particularly given the scarcity of natural predators in the area.

## 5. Conclusions

The aoudad and the Iberian ibex differ in their selection of major functional plant groups, where the aoudad tends to behave as a grazer, whereas the ibex prefers to browse from woody plants, and only under marked events of resource scarcity, either due to low plant production or a high density of herbivores, would a greater overlap in their diet be expected. Therefore, and provided sufficient resources are available, their coexistence under sympatric conditions seems to be plausible. Moreover, some evidences point to beneficial interactions in mixed groups.

Based on the results of this work and the ecological and behavioral studies previously carried out in other Iberian territories, we see no scientific basis to consider that the aoudad present in mainland Spain meets the requirements established to include it in the Spanish List of Invasive Alien Species, so we propose the withdrawal of these populations from it.

## Figures and Tables

**Table 1:** Study species presence and area covered by the different study sites.

Large Herbivores Present	Mountain Ranges	Area Covered
Iberian ibex	Mojantes	2244 ha
Aoudad	Espuña	16,277 ha
Iberian Ibex and aoudad	Burete, Quipar and Cabras	6196 ha
Iberian Ibex and aoudad	Cambrón	4906 ha

**Table 2:** Botanical composition of ibex and aoudad diet expressed as the mean percentage of epidermal fragments of woody and herbaceous plants identified in fecal samples through the microhistological technique.

Species	Distribution	Season	Woody%		Herbs%	
			Min–Max	Mean	Min–Max	Mean
Aoudad	Allopatric	Spring	27.27–37.74	32.51	62.26–72.73	67.5
Aoudad	Allopatric	Summer	51.00–71.00	60.54	29.00–49.00	39.46
Aoudad	Allopatric	Autumn	26.67–43.24	36.29	56.76–73.33	63.71
Aoudad	Allopatric	Winter	27.00–30.00	28.5	70.00–73.00	71.5
Aoudad	Sympatric	Spring	19.23–53.33	35.1	46.67–80.77	64.9
Aoudad	Sympatric	Summer	33.00–60.18	43.21	39.82–67.00	56.79
Aoudad	Sympatric	Autumn	9.35–40.20	21.31	59.80–90.65	78.69
Aoudad	Sympatric	Winter	49.00–62.00	55.5	38.00–51.00	44.5
Ibex	Allopatric	Spring	67.65–68.22	67.94	31.78–32.35	32.06
Ibex	Allopatric	Summer	66.99–78.50	71.91	21.50–33.01	28.08
Ibex	Allopatric	Autumn	43.69–76.24	56.18	23.76–56.31	43.82
Ibex	Allopatric	Winter	84.00–93.00	88.5	7.00–16.00	11.5
Ibex	Sympatric	Spring	35.34–38.39	36.86	61.61–64.66	63.14
Ibex	Sympatric	Summer	25.49–39.13	31.63	60.87–74.51	68.37
Ibex	Sympatric	Autumn	56.03–90.10	71.95	9.90–43.97	28.05
Ibex	Sympatric	Winter	33.00–48.21	39.07	51.79–67.00	60.93

**Table 3:** Botanical composition of ibex and aoudad diet expressed as the mean percentage of epidermal fragments of woody and herbaceous plants identified in fecal samples, and calibrated using the NIRS technique.

Species	Distribution	Season	Woody%		Herbs%	
			Min–Max	Mean	Min–Max	Mean
Aoudad	Allopatric	Spring	1.36–73.79	36.01	26.37–96.82	58.81
Aoudad	Allopatric	Summer	15.47–96.69	50.94	13.63–77.54	47.49
Aoudad	Allopatric	Autumn	3.65–74.27	45.19	40.3–92.12	57.78
Aoudad	Allopatric	Winter	10.24–78.15	31.84	11.66–85.19	65.98
Aoudad	Sympatric	Spring	19.71–76.05	34.59	26.86–82.44	67.08
Aoudad	Sympatric	Summer	29.73–101.19	64.1	6.99–71.15	36.71
Aoudad	Sympatric	Autumn	7.18–60.61	23.83	37.79–86	68.37
Aoudad	Sympatric	Winter	26–90.29	53.55	4.54–68	42.16
Ibex	Allopatric	Spring	7.61–88.04	65.89	4.72–62.36	27.08
Ibex	Allopatric	Summer	22.97–100.42	76.6	6.29–94.87	25.01
Ibex	Allopatric	Autumn	40.04–85.82	64.91	13.78–59.73	31.37
Ibex	Allopatric	Winter	27.4–82.58	67.27	12.98–69.13	23.72
Ibex	Sympatric	Spring	24.04–63.96	44.69	33.51–77.25	52.83
Ibex	Sympatric	Summer	35.89–82.95	61.66	18.13–66.65	39.95
Ibex	Sympatric	Autumn	20.5–93.35	62.45	6.96–76.96	35.82
Ibex	Sympatric	Winter	22.56–90.91	60.36	2.15–66.85	34.93

**Table 4:** Analysis of Variance Effects Test for the proportion of woody plants and three factors. DF = degrees of freedom.

Explanatory Variables	DF	F Ratio	Probability
Species	1	109.9875	<0.0001
Distribution	1	4.4087	0.0366
Season	3	15.6357	<0.0001
Species × Distribution	1	11.6917	0.0007
Species × Season	3	3.5585	0.0147
Distribution × Season	3	4.9621	0.0022
Species × Distribution × Season	3	8.1056	<0.0001

## Data Availability

The raw data supporting the conclusions of this article will be made available by the authors on request.
